# Bach Goes to Town †

**DOI:** 10.3390/biom8030075

**Published:** 2018-08-20

**Authors:** Jørn Bolstad Christensen

**Affiliations:** Department of Chemistry, University of Copenhagen, Thorvaldsensvej 40, DK-1871 Frederiksberg C, Denmark; jbc@chem.ku.dk; Tel.: +45-93-56-50-02

**Keywords:** Donald Tomalia, aziridines, poly(ethyleneimine), dendrimers, poly(amidoamine) (PAMAM)

## Abstract

Donald A. Tomalia is one of the pioneers in the field of dendrimers, who is still active at the age of 80 years-old. The present contribution is a journey through his scientific contributions from the early beginning until his discovery of the poly(amidoamine), (PAMAM)-dendrimers.

## 1. Introduction

Donald A. Tomalia (or just Don Tomalia in the dendrimer community) was born in the small town of Owosso, Michigan in the USA in 1938. He obtained his Bachelor (BSc) from University of Michigan and was hired by the Dow Chemical Company (Dow) in Midland, Michigan, after obtaining his Masters (MSc) from Bucknell University, Pennsylvania, under the supervision of Harold W. Heine. Later Dow financed his Doctor of Philosophy (PhD)-study under the supervision of Professor Harold Hart at Michigan State University, Michigan.

## 2. Scientific Contributions

**Scheme 1:**
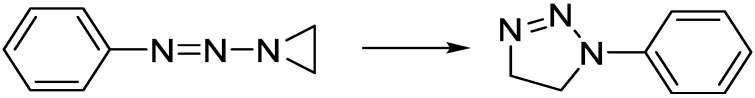
Formation of 1-phenyl-4,5-dihydro-1*H*-1,2,3-triazole.

Don was introduced to the aziridines, while working in the lab of Harold W. Heine at Bucknell University, Pennsylvania, and his first scientific publication [1] was a study on the rearrangement of *N*-aziridinyl substituted triazenes (Scheme 1).

**Scheme 2:**
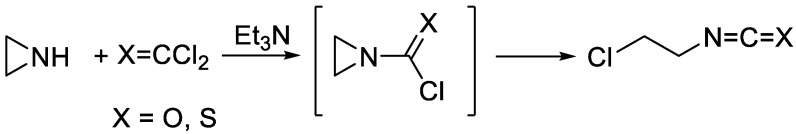
Synthesis of 2-chloroethylisocyanate and isothiocyanate from aziridine.

**Scheme 3:**
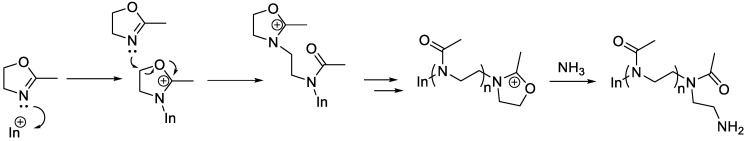
Living cationic polymerization of 2-methyl-1,3-oxazoline. In^+^ is a cationic initiator (e.g., methyl tosylate, etc.) with termination of the reaction with ammonia.

Aziridine was manufactured commercially by Dow and used as a monomer for polymers such as poly(ethylene imine). Don’s interest in aziridine chemistry led to a couple of interesting monomers [2,3,4], as shown in Scheme 2, but it was his discovery that oxazolidines underwent cationic polymerization, that was his first major achievement at Dow (Scheme 3).

The polymerization process is a living polymerization reaction, so the average molecular weight can be controlled through the ratio between the initiator and the monomer [5,6,7], and furthermore, the resulting polymer can either be functionalized at the end group by trapping with a suitable nucleophile or a new monomer can be added leading to block copolymers.

The poly(oxazolines) are converted into poly(ethyleneimine) upon hydrolysis and it is a very interesting aspect that while the polymerization of aziridine gives hyperbranched poly(ethyleneimine), the route via the poly(oxazolines) gives only the linear polymer, thereby being an early example of the ability to control polymer architecture.

**Scheme 4:**
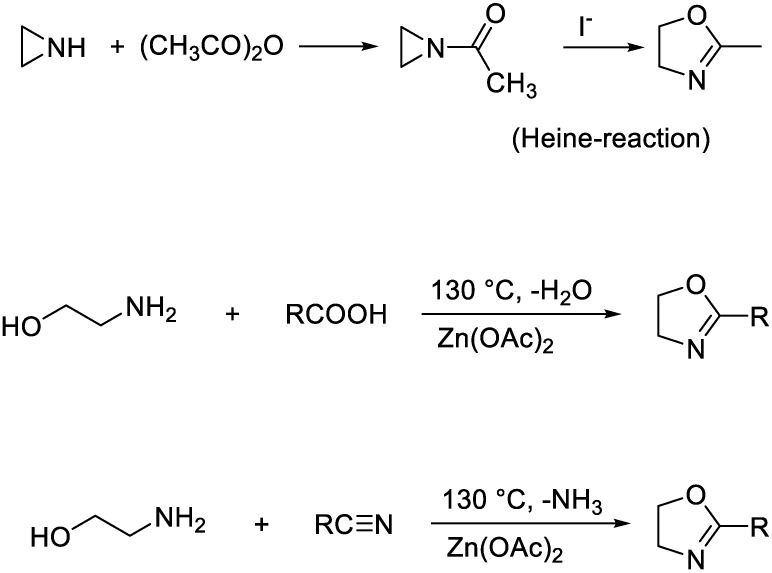
Three different methods for synthesizing 2-alkyl-1,3-oxazolines.

Oxazolines are available by a number of methods including the Heine reaction (Scheme 4).

**Scheme 5:**
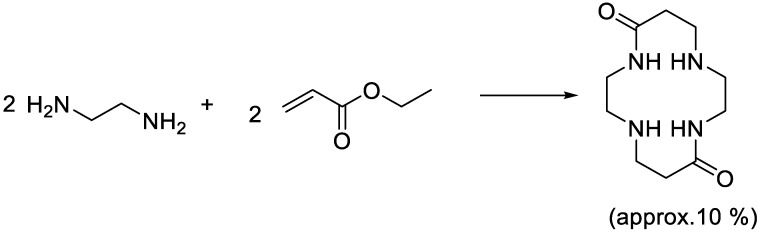
The simple synthesis of 1,4,8,11-tetraazacyclotetradecane-5,12-dione from ethylenediamine and ethyl acrylate.

The next important discovery was that the reaction between ethylenediamine and methyl acrylate (both large scale products of Dow) could give well-defined compounds and not just polymers as claimed in previous patents [8]. A patent issued in 1986 describes the formation of a macrocyclic amide by the reaction between ethylenediamine and ethyl acrylate [9] (Scheme 5), and although the yield is modest, it is surprising that the compound is formed at all.

**Scheme 6:**
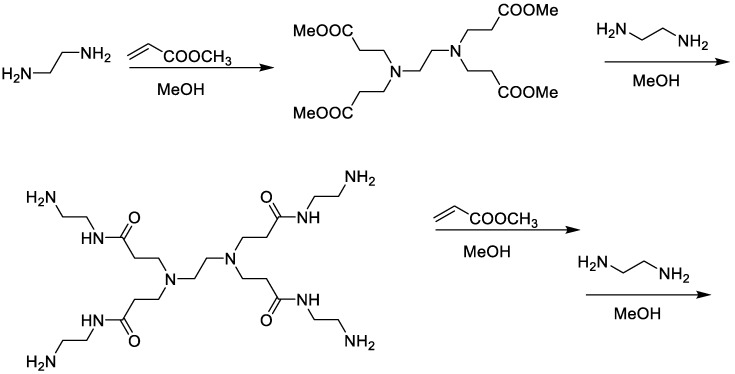
The synthesis scheme for synthesizing ethylenediamine core poly(amidoamine) (PAMAM)-dendrimers.

And finally, in 1984, the first patent [10] on poly(amidoamine) (PAMAM)-dendrimers, followed by the first papers in 1985 [11] and 1986 [12] which made Don famous (Scheme 6).

The name dendrimers was invented by Don [13] and is derived from the Greek word for tree, but the concept of dendritic growth was not new and had been around for a long time; it is found many places in nature and the polymer theoretician Flory [14] suggested this type of polymer architecture. Vögtle and Buhleier [15] described cascade molecules in a paper from 1978 that were in fact low generation poly(propylene imine) (PPI)-dendrimers. In 1979 Denkewalter and coworkers [16] filed a patent application on “Macromolecular highly branched homogeneous compound based on lysine units” which in reality is lysine-dendrimers. The patent was granted in 1981.

Don joined Michigan Molecular Institute in 1990, cofounded Dendritech in 1992, Dendritic NanoTechnologies in 2002, and in 2010 NanoSynthons, which he is the currently running.

## 3. Bach Goes to Town

**Figure 1:**
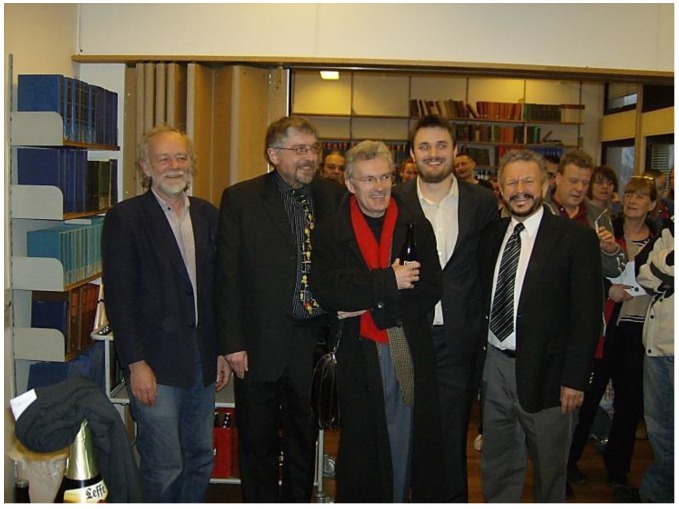
Relaxing after the PhD-defense. From left to right: Klaus Bechgaard (chairman of the committee), the author (supervisor), David Tanner (external member), Michael Pittelkow (the PhD student) and Don.

In November 2006, one of my PhD-students was handing in his thesis on the topic of dendrimers and we needed an external non-Danish national as one of the members of the evaluation committee. I asked the student and he suggested that we could ask Don and my response was something like: “We can ask him, but he is probably way too busy”. But to our surprise, he wrote back and said that he would very much like to come to Copenhagen to take part in the defense (which took place in February 2007). Don came to Copenhagen and turned out to be a really nice person! He was introduced to the rest of the group as well as to my collaborators Peter Heegaard and Ulrik Boas, with whom I had coauthored the first book to bridge the gap between biology, medicine, and dendrimers (Dendrimers in Medicine and Biotechnology), which had been published in 2006 [17] by Royal Society of Chemistry (RSC) Publishing (we had basically tried to write the book that we would have wanted, when we entered the field and from the responses we got, it seemed that it filled a need). It turned out that Don was one of our fans and we all had a very enjoyable time discussing a lot of chemistry (Figure 1).

Later that year was the 5th International Dendrimer Symposium (IDS-5), which was organized by Jean-Pierre Majoral, Anne-Marie Caminade, and coworkers in Toulouse, France. I went there together with Ulrik Boas and as soon as Don spots us, he told us about an upcoming book to be published by Cambridge University Press that he wanted Ulrik Boas and I to coauthor with him and next he asked me, when I would be coming to the USA to visit him in Michigan!

I went for a sabbatical in 2008 to University of Central Michigan in Mount Pleasant, Michigan, where Don had a professorship at that time with the plan that I should be able to finish my contributions to the upcoming book while I was there. It was a very productive stay although we did not finish the book—it was published in 2012 [13], but I got involved in a lot of other activities and became a part of the extended Tomalia-family. The following years, I usually spend part of my summer with Don, family, and coworkers. I especially remember searching through a warehouse full of second hand equipment for sale at Dow together with Don finding hoods and lab equipment for NanoSynthons ending up getting the items for a very reasonable price including a pallet of A4 printing paper bought by mistake (the standard format in the USA is US letter) and a lot of brand new glass containers for samples. Don not forget! Don is a very clever merchant always looking for a good deal! The following year, I helped organize the chemical inventory at the company besides discussing the concept of nanoperiodicity and different ways to classify nanomaterials [13,18,19]. I could tell many more stories about wonderful times in Michigan, but that will be another time, another place.

## 4. Discussion

So where is the field of dendrimers now with respect to biomedical applications? The first dendrimer-based drug Vivagel^®^ is now being approved around the world. It is a surface modified lysine-dendrimer for topical use for prevention of sexually transmitted disease developed by the Australian company Starpharma LLC, which also claim to have more dendrimer-based drug compositions under development [20]. IMD-Pharma in France is working on the use of Majoral-Caminade-phosphorous dendrimers for treatment of inflammatory diseases [21]. There are also several dendrimer-based drugs or delivery systems under development in labs from academia, but the major problem is the lack of facilities (small-medium enterprises (SMEs) or University) with the capability of synthesizing dendrimers under good manufacturing practices (GMP)-conditions. A major problem is that most dendrimers are made by classical organic synthesis but the purification and characterization requires methods that are common to biotechnology, and the vast majority of GMP-companies are either making small molecules by organic synthesis or making biological macromolecules by biological methods. Furthermore, expanding the capacity of a small molecule GMP-manufacturer (which is well-equipped for doing synthesis) into making dendrimers requires significant investments in equipment and validation and this is at the present considered too risky by many producers. Of course that will eventually change as a market emerges, but at the present it is a show-stopper.

## 5. Conclusions

Dendrimers must at the present be considered a technology platform for medicine just like liposomes, but we are only at the early beginning in translating the findings from bench to bedside. People like Don have been and are invaluable in spreading the gospel of dendrimers to the world outside the labs, and we need more people like that!

The choice of title for this birthday story was not random; Don likes classical music very much—especially W. A. Mozart and he is very good at recognizing patterns in scientific observations. There is a clear path from the discovery of linear poly(ethyleneimine) versus hyperbranched poly(ethyleneimine) via the simple synthesis of 1,4,8,11-tetraazacyclotetradecane-5,12-dione (Scheme 5) from acrylic acid esters and ethylenediamine to the PAMAM-dendrimers and into the concept of nanoperiodicity. J. S. Bach composed highly structured music and was expected to deliver new compositions on a regular basis—while at Dow Don was expected to deliver new commercially interesting molecules and polymers on a regular basis. However, I do not find it likely, that Don had discovered the PAMAM-dendrimers if he had been as structured as Bach. He is more like a Mozart playing with chemistry or like a jazz-influenced Bach in that respect and hence the title.

I was in Michigan when Don turned 70 and although it seems like yesterday it is 80 years-old this time. Dear Don, I wish you a happy birthday and I hope you will remain productive for many years to come!
